# Bovine Decellularized Amniotic Membrane: Extracellular Matrix as Scaffold for Mammalian Skin

**DOI:** 10.3390/polym12030590

**Published:** 2020-03-05

**Authors:** Andrea Catalina Villamil Ballesteros, Hugo Ramiro Segura Puello, Jorge Andres Lopez-Garcia, Andres Bernal-Ballen, Diana Lorena Nieto Mosquera, Diana Milena Muñoz Forero, Juan Sebastián Segura Charry, Yuli Alexandra Neira Bejarano

**Affiliations:** 1 Laboratorio de Investigaciones en Salud, Universidad Manuela Beltrán, Avenida Circunvalar No. 60-00, Bogotá 110231, Colombia; hugo.segura@umb.edu.co (H.R.S.P.); lorena.nieto@umb.edu.co (D.L.N.M.); diana.munoz@umb.edu.co (D.M.M.F.); reumatologiasegura@hotmail.com (J.S.S.C.); yuli.neira@umb.edu.co (Y.A.N.B.); 2 Centre of Polymer Systems, University Institute, Tomas Bata University in Zlín, Trida Tomase Bati 5678, 76001 Zlín, Czech Republic; vextropk@gmail.com; 3 Grupo de Investigación en Ingeniería Biomédica, Vicerrectoría de Investigaciones, Universidad Manuela Beltrán, Avenida Circunvalar No. 60-00, Bogotá 110231, Colombia; andres.bernal@docentes.umb.edu.co

**Keywords:** decellularization, biological scaffolding, bovine amniotic membrane, extracellular matrix, tissue regeneration

## Abstract

Decellularized membranes (DM) were obtained from bovine amniotic membranes (BAM) using four different decellularization protocols, based on physical, chemical, and mechanical treatment. The new material was used as a biological scaffold for in vitro skin cell culture. The DM were characterized using hematoxylin-eosin assay, scanning electron microscopy (SEM), Fourier transform infrared spectroscopy (FTIR-ATR), and differential scanning calorimetry (DSC). The in vitro cytotoxicity of DM was evaluated using MTT. The efficacy of decellularization process was assessed through DNA quantification and electrophoresis. All the used protocols showed a high effectiveness in terms of elimination of native cells, confirmed by DNA extraction and quantification, electrophoresis, and SEM, although protocol IV removes the cellular contents and preserve the native extracellular matrix (ECM) architecture which it can be considered as the most effective in terms of decellularization. FTIR-ATR and DSC on the other hand, revealed the effects of decellularization on the biochemical composition of the matrices. There was no cytotoxicity and the biological matrices obtained were a source of collagen for recellularization. The matrices of protocols I, II, and III were degraded at day 21 of cell culture, forming a gel. The biocompatibility in vitro was demonstrated; hence these matrices may be deemed as potential scaffold for epithelial tissue regeneration.

## 1. Introduction 

Tissue engineering aims to regenerate damaged tissues, developing biological substitutes which along with a thriving cell growth, may restore, maintain, or improve a functional tissue [1,2,3]. This field has undergone rapid development in the last quarter of the twentieth century, although this science is devoted to skin regeneration it is still a major scientific and clinical challenge [4,5], and the healing response to chronic wounds is poorly understood and a matter of debate [6]. The skin can be considered as the largest organ, which covers the entire surface of the body and its main function is to serve as protective barrier against chemical, mechanical, and infectious damage. Nonetheless, injuries from trauma or skin-burns result in large-scale tissue loss, therefore, autografts, allografts, and xenografts are traditionally used. However, these kinds of treatments have limitations, such as immune rejection, and primary contraction [7].

Ideal skin substitutes should mimic the natural functions of the skin and the structural properties of the extracellular matrix; moreover, it has to protect the organism from protein loss, and it should improve the aesthetic appearance of the wound as well as inhibit the growth of exogenous microorganisms [7,8].

As an innovative treatment for skin injuries, biological substitutes have appeared, and they have the function of supporting growth, differentiation, and cell migration, and may come from different substrates either natural or synthetic, such as, collagen, gelatin, hyaluronic acid, fibronectin/fibrin, chitosan, alginate, polyglycolic acid, polylactic acid, and polycaprolactone; likewise, biological scaffolds composed of extracellular matrix (ECM) of decellularized tissues may be also used as biological substitutes [9,10,11,12,13]. Within this context, decellularized tissues and organs have successfully been used in a variety of tissue engineering/regenerative medicine applications, and the used decellularization methods vary as widely as the tissues and organs of interest [14]. The importance of ECM stems from its three-dimensional ultrastructure and its composition provides a microenvironment that guides the organization, growth, and differentiation of skin cells [15,16,17,18,19,20,21]. From all the mentioned substrates, collagen as a part of the ECM has been extensively employed as a biomaterial in cellular therapies and tissue engineering [14,21,22,23,24], and its relevance as a candidate for tissue engineering has been described in great extent [25,26].

Despite of the recent breakthroughs in terms of tissue regeneration, wound healing is a complex process that involves activation and synchronization of intracellular, intercellular, and extracellular mechanisms, including coagulation and inflammatory events, fibrous tissue accumulation, collagen deposition, epithelialization, contraction of the wound, tissue granulation, and remodeling [14,15,27,28]. Therefore, grafting materials must exhibit biodegradable, biocompatible, and adequate mechanical properties as well as support normal tissue regeneration [3,29]. In that regard, skin substitutes for wound healing from biological materials based on animal ECM have been developed and the decellularization process has reached an important level of success [14,15,30,31,32,33]. Over the years, xenografts have been obtained from various animal species, including birds, rodents, felines, canines, bovines, and swine [34,35]. To this day, the available acellular ECM scaffolds include swine and bovine equine substrates as well as human amniotic membranes [14,15,30,35,36,37,38,39,40].

Unfortunately, despite the numerous investigations in this area, clinical wound treatment remains unsatisfactory in many cases [15]; chronic wounds require long term and intensive care, and the associated cost are high [31]. Specialists agree that there is still no ideal skin substitute available [31,41,42] and the high costs and time required for the preparation of biological substitutes are crucial factors for developing new materials [35]. Moreover, the risk of zoonotic infections that might be transferred from the graft to the patient is latent, and allergic reactions is the main contraindication for using these kinds of materials [3,34,43]. On the other hand, the human amniotic membrane presents relevant disadvantages, such as that it is scarce due to its high cost, it has poor mechanical properties, and it may be a source of infectious diseases spreading [44,45].

The need for skin substitutes is of paramount importance specifically for large defects of burns, congenital diseases, traumas, and infections [46]. In this frame, acellular amniotic membrane might have potential as a matrix for tissue regeneration or as a substrate to facilitate autologous/allogeneic cell transfer [47]. Human decellularized amniotic membrane has been widely shown as a biodegradable and bioactive matrix for regenerative tissue repair [48]. Thus, this study proposes a candidate that brings the inherent attributes of bovine amniotic membranes (BAM), which can be useful for being used as a matrix for skin regeneration. For this purpose, four distinct protocols were proved (details are shown in methodology section). The obtained decellularized membranes (DM) were used as an alternative scaffold for skin regeneration, and as a sources of collagen IV and VII, elastin, laminin one and five, fibronectin, and entactin [46,49,50]. This material has similar properties to other matrixes obtained from skin [51,52]. Therefore, DM were obtained from four different methods and the efficiency of these methods were evaluated. The cell cultures were carried out on samples and there is evidence of the material does not behave cytotoxically. The obtained DM demonstrated potential in the use of skin regeneration, which is a valuable alternative for tissue engineering and the prospectives of its applications are a new challenge in the field of biomaterial science.

## 2. Materials and Methods

### 2.1. Decellularization of the BAM

The bovine amniotic membrane was obtained from a vaginal birth of a bovine female with no infectious-contagious diseases within aseptic conditions. Samples were collected and transported at 4 °C in centrifuge tubes containing a solution of phosphate buffered saline (PBS) that included antibiotics (penicillin, streptomycin, and amphotericin B). Thereupon, the samples were washed with cold PBS and dissected in sections of 16 cm^2^ sections in the biological class II biosafety cabinet (ESCO, IDN).

Four protocols were used to decellularize the BAM (Table 1). A control sample was kept without frozen treatment at −20 °C. For protocols I, II, and III, the separation of two layers, fetal and maternal, was performed as it is reported in most of the investigations [52,53,54,55]. On the other hand, this procedure was not performed in protocol IV, in order to observe variations in the properties of the membrane regarding exposure with the chemical solutions used and in cell culture.

All BAM were subjected to a freezing cycle in liquid nitrogen (−196 °C) for 22 h and unfreezing in a serological bath (Polyscience, Niles, Illinois, USA) at 37 °C for two hours. Then, BAM were treated with strong and weak detergents (sodium dodecyl sulphate (SDS) 0.1% or Tween 80) for 4 h followed by being soaked in a base solution (NaOH 0.1 M) for 1 h and acid solution (peracetic acid (PAA) and ascorbic acid or ethanol). After, as a final wash, ethanol at 70% was applied for 1 h to remove residual nucleic acids and phospholipids from the tissue and finally, PBS as a buffer solution was pertained for 2 h. The membranes were mechanically stirred throughout the process using an orbital shaker (Camlab, Cambridge, UK) to ensure a homogeneous wash and a minimal damage to the tissue ultrastructure [5,6,7,30].

Once the abovementioned process was finished, protocols II and IV needed a new acid/basic treatment in order to wipe out the remains of color in the membranes; therefore, it was necessary to immerse them again in NaOH for one hour and PAA for another hour. For BAM treated in protocols I and III, it was not necessary to carry out more washings, and samples were stirred in ethanol at 70%.

After each decellularization step, BAM were washed with deionized water for 30 min in a shaker to eliminate tissue remnants and the used substances. Finally, all the membranes were washed four times with PBS for 30 min and stored at −22 °C.

### 2.2. Determination of DNA Content

#### 2.2.1. Extraction of DNA

To ensure the removal of all cellular and nuclear material in the decellularized BAM, the DNA extraction process was carried out using a PureLink^®^ kit (Invitrogen). <25 mg of BAM and DM were placed into a micro centrifuge tube. It was added to 180 μL of genomic digestion buffer and 20 μL of proteinase K to remove lipids and digest proteins. Then, the treated samples were incubated at 55 °C in a serological bath with vortex every 10 min for one hour and centrifuged at 13,000 rpm for 3 min at room temperature. Each supernatant was transferred to a new sterile micro centrifuge tube and 20 μL of RNase A was added, mixed, and incubated for two minutes. Subsequently, 200 μL of lysis buffer and 200 μL of 99.9% ethanol were added to each lysate to precipitate the DNA by vortexing for five seconds.

Once the DNA was extracted, it was purified by a series of washes, placing the previous preparations in collector tubes with a column and centrifuging at 12,000 rpm for one minute. Washes were carried out with 500 μL of wash buffer, 1500 μL of wash buffer two, and 50 μL of buffer elution, centrifuging after each addition for one, two, and three minutes, respectively. Finally, the microcentrifuge tubes containing DNA were stored at 4 °C.

In the wells of an agarose gel, samples of the extracted DNA were placed in the horizontal electrophoresis chamber (Thermo EC, Holbrook, NY, USA). Afterwards, the movement of the bands was observed in the transilluminator (Fisher Biotech, Pittsburgh, PA, USA).

#### 2.2.2. DNA Quantification

DNA concentrations were obtained using a QuantiFluor^®^ dsDNA System Kit (Promega, Madison, Wisconsin, USA). After the DNA extraction, the DNA samples were prepared by the addition of 1–20 µL to 200 µL of working solution in 0.5 mL PCR tubes and vortexing, and incubated at room temperature for five minutes, in a dark condition. Finally, fluorescence was measured in the calibrated Quantus™ fluorometer (Promega, Madison, Wisconsin, USA). The effectiveness of each decellularization protocol was evaluated by triplicated. 

### 2.3. Cell Culture 

Cells were obtained from a full thickness ovine skin biopsy and cultured with RPMI-1640 culture medium, supplemented with 5% fetal bovine serum (SFB) and 1% antibiotic penicillin, streptomycin, and amphotericin B (Sigma-Aldrich, Bogota, Colombia) in an incubator at 37 °C in a humidified atmosphere of 5% CO_2_. Monitoring culture was carried out every three days with an inverted microscope (Olympus, New York, NY, USA). After cell confluence, cells were sub-cultured through trypsinization and used in the second pass.

#### Cell Seeding on DM and 3-(4,5-d imethylthiazol-2-yl)-2,5-diphenyltetrazoliumbromide (MTT) Assay

The in vitro cytotoxicity of DM was evaluated using 3-(4,5-dimethylthiazol-2-yl)-2,5-diphenyltetrazoliumbromide (MTT) assay (Cell Biolabs Inc., Bogota, Colombia). Specimens of 10 mm in diameter were cut and placed at the bottom of a 24-well-plate (Corning, New York, NY, USA). Before the culture, samples underwent a sterilization process with ultraviolet light for 15 min each side. Then, cells were seeded on DM at a density 5 × 10^4^ cells/well in the previous mentioned incubation conditions.

After culturing for 24, 48, and 72 h, 50 μL of the CytoSelect™ MTT Cell Proliferation Assay Reagent was added to each well and incubated for 4 h, until purple precipitate was visible. Then, 500 μL detergent solution was added and incubated at room temperature for two hours. A specific culture media (RPMI) was also considered as control. The absorbance of solution was measured using a microplate reader 800 ^TS^ (Biotek, Winousky, Vermont, USA) at 490 nm. Cell viability was determined using Equation (1). For histological analysis with hematoxylin-eosin, a DM sample was cultured until day 21.

Cell viability (%) = Absorbance sample/Absorbance control (untreated) × 100(1)

### 2.4. Histological Analysis with Hematoxylin-Eosin 

Control sample BAM, DM, and recellularized DM (at 21 days culture) tissues were fixed in 10% formaldehyde, embedded in paraffin, cut into sections of 5 μm, stained with hematoxylin-eosin, and observed under the optical microscope (Olympus, Tokyo, Japan) to evaluate the presence of nuclear material.

### 2.5. Scanning Electron Microscopy (SEM)

Micrographs of the prepared samples were taken by the scanning electron microscope Nova NanoSEM 450 (FEI, Brno, Czech Republic) with a Schottky field emission electron source operated at an acceleration voltage ranging from 200 V to 30 kV and a low-vacuum SED (LVD) detector. A coating with a thin layer of gold was performed by a sputter coater SC 7640 (Quorum Technologies, Newhaven, East Sussex, UK).

### 2.6. FTIR-ATR Spectroscopy

FITR spectroscopy analysis was carried out on NICOLET 6700 FTIR spectrometer device (Thermo Scientific, Waltham, MA, USA) equipped with attenuated total reflectance (ATR) accessory utilizing the Zn–Se crystal and software package OMNIC over the range of wavelengths from 4000 to 600 cm^−1^ at room temperature under a resolution of 4 cm^−1^. Each spectrum represents 64 co-added scans referenced against an empty ATR cell spectrum.

### 2.7. Differential Scanning Calorimetry (DSC)

Calorimetric measurements were carried out in a differential scanning calorimetry (DSC) 1 calorimeter, Mettler Toledo (Greifensee, Zurich, Switzerland), under nitrogen flowing at a rate of 30 mL min^−1^. The specimens were pressed in sealed aluminum pans. A heating cycle was performed in order to acquire the glass transition temperature (T*_g_*) and melting temperature (T*_m_*). The samples were cooled down by nitrogen at an exponentially decreasing rate. The heating of the cycle was performed from 25 to 240 °C at a rate of 20 °C/min. The T*_g_* was determined as the midpoint temperature by standard extrapolation of the linear part of DSC curves using Mettler-Toledo Stare software and the T*_m_* as the maximum value of the melting peak.

### 2.8. Statistic Analysis 

MTT measurements were performed in triplicate. All experimental values were expressed in form of average ± standard deviation. Results were statistically compared using one-way analysis of variance (ANOVA) with *p* < 0.05.

## 3. Results and Discussion

### 3.1. Decellularization of BAM and DNA Content

**Figure 1:**
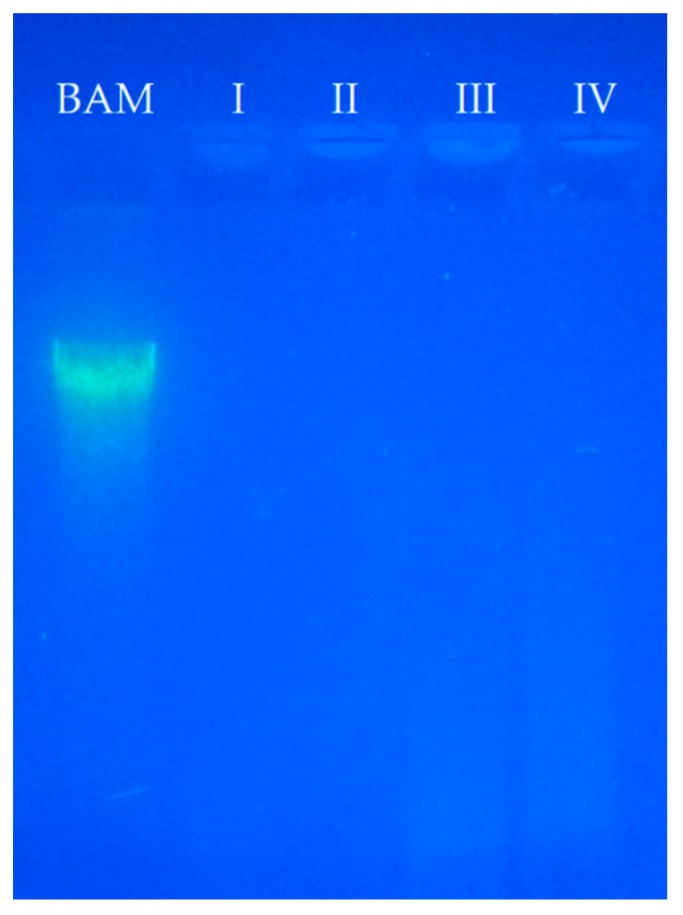
Electrophoresis pattern obtained in agarose gel for the used protocols (I, II, III, and IV), and for the control sample (BAM).

The main purpose of decellularization of xenogeneic matrices is to effectively eliminate cells and nucleic acid residues, as well as preserve the composition of the ECM [11]. In this frame, the DNA content analysis in DM indicated total cell absence whereas in BAM is easily observable (Figure 1). Moreover, the four protocols achieve cell removal until the detection limit of the test.

**Figure 2:**
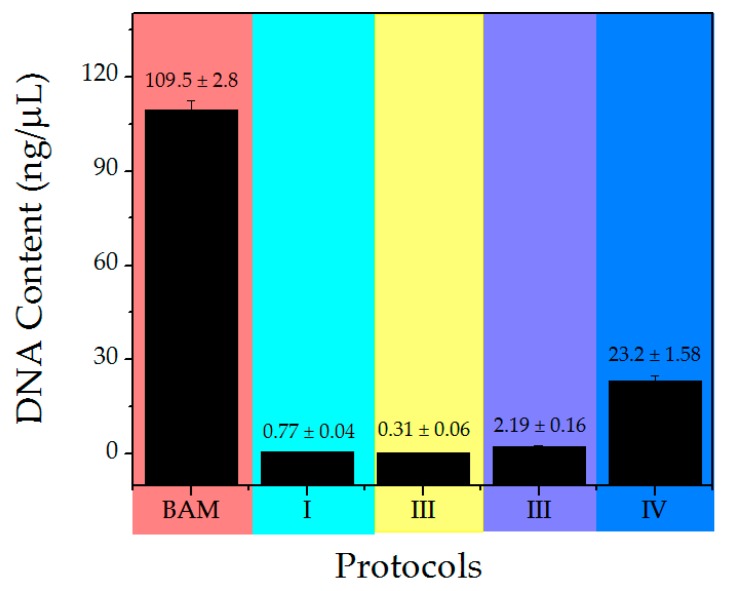
DNA content obtained for the control sample and decellularized membranes (DM).

In the electrophoresis technique, the DNA moieties are so small that they cannot be observed while they migrate through the gel, as it is possible in the control membrane (BAM). This technique allowed the separation, identification and isolation of DNA fragments, which cannot be separated by other methods. However, the quantification of DNA allows for the detection of small amounts of the nucleic acid, that is, the actual value of respective DNA for each protocol. From smallest to largest value, so it is highly sensitive. DNA content analysis of DM was conducted to compare the efficiency of the previously developed decellularization protocols. The obtained results showed that the DNA levels decreased with each protocol in comparison to BAM (Figure 2). ANOVA test showed that no significant differences were evidenced in the tested protocols. It has been reported that a lower concentration of <50 ng/mg in a membrane implies that the matrix can be considered as decellularized [32,56]. Therefore, the chemical and mechanical methods used were effective at eliminating the DNA content from the DM. Other studies have shown that higher degrees of decellularization measured by DNA content are associated to a better tissue remodeling in vivo and macroscopic response in the host [57,58]; therefore, protocol II would be considered as the most suitable for decellularization process.

The decellularization protocols consisted of the application of physical freeze-unfreeze method to lyse cells through the formation of microcrystals. This technique requires smaller amounts of chemical agents, which do not significantly alter the ECM properties [10,59,60]. With liquid nitrogen, a lower number of cycles and shorter time were required compared to freezing-unfreeze protocol at −20 or −80 °C [60].

Sodium dodecyl sulphate used as an ionic detergent has the ability to efficiently remove cells and genetic material [58,61] as it was observed in the electrophoresis and confirmed by the DNA content of protocols I and II. Likewise, SDS contributes to the inhibition of collagen calcification processes [62]. However, it can alter the ultrastructure and the elimination of growth factors [58,59,61]. Tween-80 is a non-ionic detergent, considered mild, that has the property of solubilizing proteins while maintaining the structure of the native protein [10]. In the electrophoresis of samples prepared using protocols III and IV, a slight sweep was observed due to protein residues most likely associated with the use of this detergent and DNA content analysis corroborated the presence of DNA in low concentration after decellularization. DM from protocol IV contained double layer (amnion and chorion), therefore, the surface area of exposure to chemical agents was smaller and consisted of even more DNA residues. For this reason, it contains more DNA; however, the obtained value for this protocol is lower in comparison to the reported value of <50 ng/mg for a membrane which is considered decellularized.

It is imperative to emphasize that the use of alkaline or acidic solutions in excess may cause serious alteration on the ECM [10]. Alkaline solutions denature chromosomal DNA and plasmid; however, they degrade collagen to a certain extent and eliminate growth factors from the resulting DM and reducing its mechanical properties. The exposure of the matrices to NaOH was performed for one hour, since a prolonged exposure may disintegrate the tissues and interrupt the formation of collagen crosslinks [3]. On the other hand, acids dissociate the DNA of the ECMs via solubilization of cytoplasmic components and the disruption of nucleic acids. PAA with hydrogen peroxide or ethanol was effective for the disinfection and removal of cellular debris from the BAM [3]; ethanol was used for the final wash to eliminate the residual nucleic acids, and delipidize the tissues in addition to its microbicide action, necessary for the manipulation to which the membranes were exposed [10]. Washes with PBS were indeed effective to remove chemical traces and to neutralize the pH of the samples for cell culture. Cells need strict culture conditions to survive, and variation in those conditions can trigger apoptosis [63].

In comparison to other human amniotic membrane decellularization studies, no antibiotics or enzymes were used, which are usually associated with bacterial resistance and irreparable damage to the matrices [53]. Finally, the low obtained standard deviation in this process is an indicative of the reproducibility of the decellularization protocols.

### 3.2. MTT Assay

**Figure 3:**
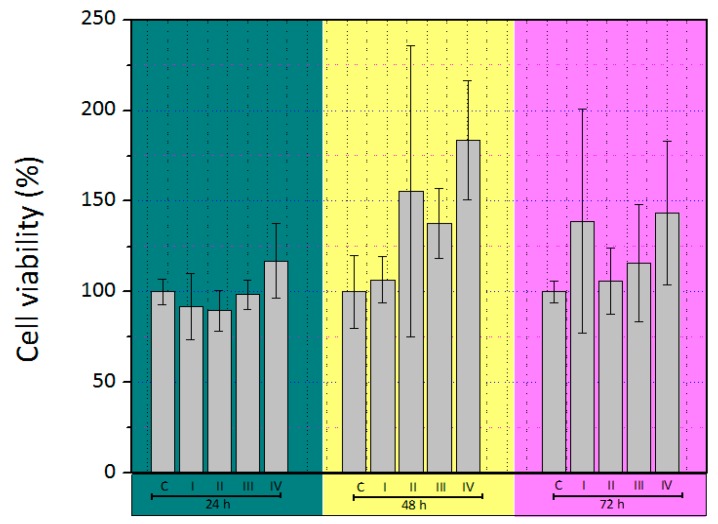
3-(4,5-dimethylthiazol-2-yl)-2,5-diphenyltetrazoliumbromide (MTT) assay of skin cells growing on different DM for 24, 48, and 72 h. * *p* < 0.05.

The viability of skin cells seeded on DM of different protocols was measured in terms of cellular mitochondrial dehydrogenase activity using MTT assay. The viability of seeded cells for 24, 48, and 72 h are depicted in Figure 3. It was observed that the cells sustained their metabolic activity in culture on the DM, and that activity was increased during the time, showing a considerable biocompatibility of DM. At 72 h, the activity was not observable as a consequence of the detergent did not longer dissolve MTT.

The obtained data underwent ANOVA testing and the results indicated that no significant differences were evidenced in all the tested protocols. This was likely due to the nature of DM and and its composition (mainly collagen). Moreover, it was revealed that detergent, acids, and bases removal are critical for generating optimal acellular scaffolds with potencial clínical uses. In this way, any of the tested protocols show cytotoxic effects on the seeded cells. The cell number augmented with increasing the incubation time, which is an indicator for improving the effect of DM on the metabolic activity of the cells compared to a control culture. DM of protocol IV exhibited a visible increase in metabolic activity, associated to the fact that it retained its biochemical properties to a greater extent. These results are in a good agreement with other reports which indicate that scaffolds made of decellularized amniotic membrane, did not exhibit cytotoxicity [64].

The results of the previous studies suggest that the vast majority of current decellularization protocols are detergent-based and incompletely removed residual detergents may have a deleterious impact on subsequent scaffold recellularization [10,29,58,65]. Residual SDS within biomaterials has severe cellular toxicity and may be responsible for the decrease in cell growth [10,29]. Therefore, the success of subsequent recellularization is based on the removal of the lysed cellular material and cytotoxic detergent after the decellularization process [65].

**Figure 4:**
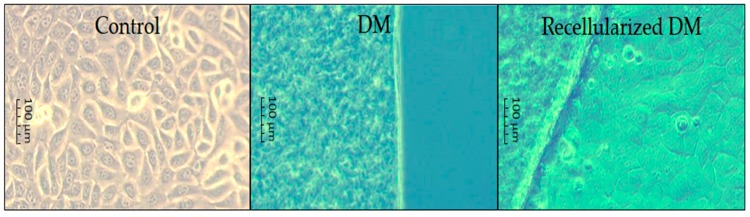
Images of cell culture on DM for mammalian skin cells obtained using an inverted microscope (40×).

The progress of cell cultures is shown in Figure 4. On day 21 in protocols I, II, and III, degradation was observed and the membrane of protocol IV remained intact. Furthermore, protocols I, II, and III were degraded and it was not possible to carry out histological analysis. The histological findings corroborated the cell growth on the DM.

### 3.3. Histological Analysis 

**Figure 5:**
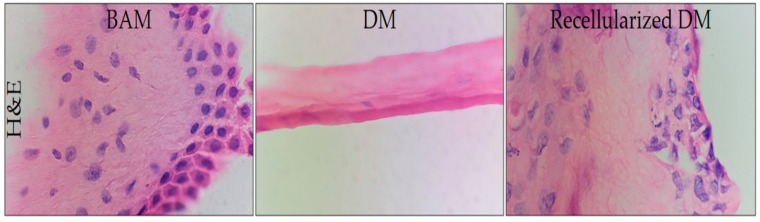
Microphotographs for BAM (**left**), DM (**middle**), and recellularized DM (**right**) obtained from the hematoxylin-eosin (H&E) assay (100×).

In the histological study of BAM, a simple cubic epithelium was observed (Figure 5) that included large binucleated (basophilic) cells and native collagenous (eosinophilic) fibers [49,54] of normal bovine tissue. In this technique, the efficiency of the decellularization protocols was substantiated by cellular absence. Hematoxylin-eosin assay (H&E) disclosed abundant mammalian skin cells adhered to the recellularized BAM as it was observed during cell culture monitoring until day 21. The microphotographs are shown in Figure 5. No cells were observed in the DM as a consequence of the acidophilic matrix. Moreover, cells were present after 21 days of culture in the recellularized DM.

Although several studies recommend the use of cell lines in this kind of experiments [66,67,68,69,70,71], it is of paramount importance to indicate that in vitro studies have evidenced that in a standard cell culture, fibroblast positively influence keratinocyte growth, most likely due to the fact that these cells secrete soluble growth factors. In natural skin, the interaction is relevant as well. Without fibroblasts, the keratinocyte differentiation is severely affected. Moreover, keratinocytes have also a positive effect on the proliferation of fibroblasts. Based on these findings, it is possible to affirm that in order to gain meaningful data from toxicological in vitro studies, the isolated focus on a keratinocyte-containing epidermal layer alone is not sufficient, making the use of a full-thickness skin model essential [14,72,73,74].

The amniotic membrane has structures, which are histologically similar to the skin, i.e., composed of a multilayer epithelium and the basic membrane, and the structure might be considered as a good support for wound healing, reepithelialization and inhibition of scar formation and bacterial growth [50,52,75,76].

### 3.4. Scanning Electron Microscopy

**Figure 6:**
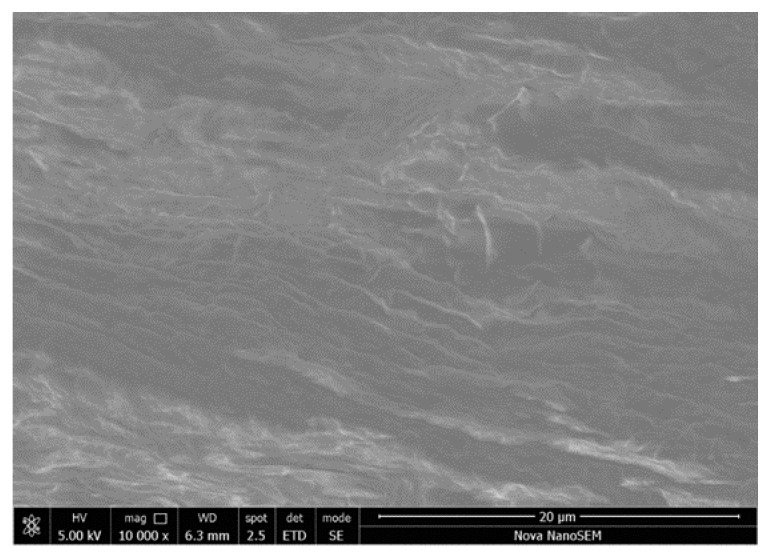
SEM image for BAM.

Topographical analysis shows that the native BAM contained collagen fibers with tissue cells on an irregular surface (Figure 6). This result is in a good agreement with the obtained by electrophoresis and histology studies, where the control membranes presented the DNA band.

**Figure 7:**
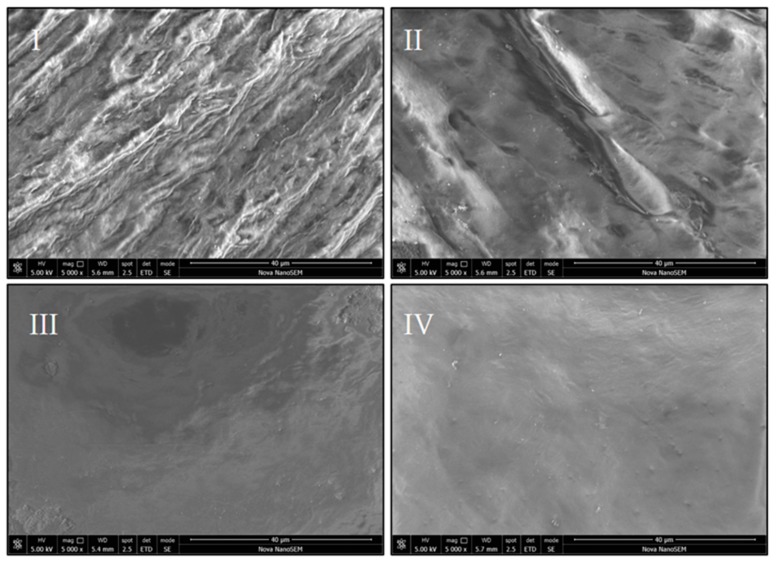
SEM images for DM obtained from the tested protocols.

The micrographs of the studied membranes also confirm that the processes were efficacious for the elimination of the cells in all the tested protocols. There are differences in the surface of each membrane; for instance, image from protocol I depicts a surface where the collagen fibers are very similar to the native ones, whereas DM for protocol II is a smoother surface. DM obtained using protocol III showed tissue wear along with some crystalline residues and the sample of protocol IV is the most homogeneous of the appraised surfaces (Figure 7).

### 3.5. FTIR-ATR Spectroscopy

**Figure 8:**
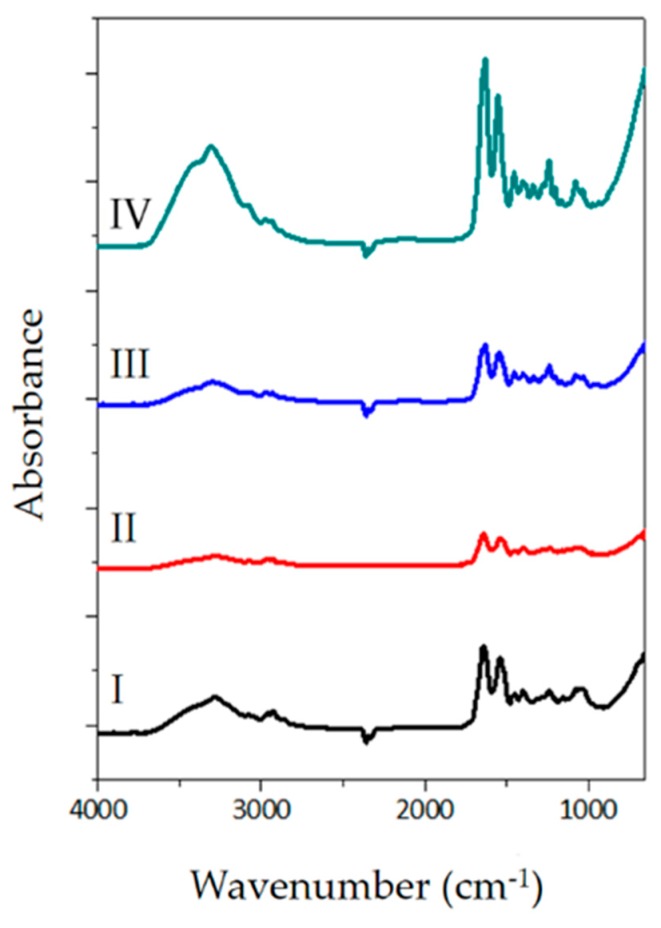
FTIR spectra for DM obtained from the four protocols.

Attenuated total reflectance Fourier transform infrared (ATR-FTIR) spectra of the assessed samples are shown in Figure 8. The peptide characteristic bands at approximately 3300, 3000, 1630, 1545, 1240, and 690 cm^−1^ are identifiable. For example, the amide I is a broad band around 1640–1630 cm^−1^ originated from C=O stretching vibrations coupled to N–H bending vibration. The amide II band, which is located at around 1550 cm^−1^ arises from N–H bending vibrations coupled to C–N stretching vibrations. Finally, the amide III characteristics bands, that usually appear within the range of 1300–1200 cm^−1^ result from the interaction between N–H bending and C–N stretching. The band locate at 690 cm^−1^ is an usual amide vibration which emerges from out of plane N–H wagging [77,78,79,80].

The absorption peaks within the 3000–2800 cm^−1^ spectral range are attributed to aliphatic C–H stretching; on the other hand, the bands around 1500 cm^−1^ are associated with C–H bending. The studied spectra possess the typical features of collagen-like proteins, which have been extensively studied in previous scientific works [77,78,81]. Nevertheless, the characteristic collagen bands are visible, which implies that collagen is retained upon each decellularization process, there are visible differences in the intensity of spectral bands, which may be ascribed to the interaction of the membranes with the solutions, the duration, and harshness of each decellularization protocol. In fact, there is a triple helix denaturation, which may be evinced on the intensity bands.

### 3.6. Differential Scanning Calorimetry (DSC)

**Figure 9:**
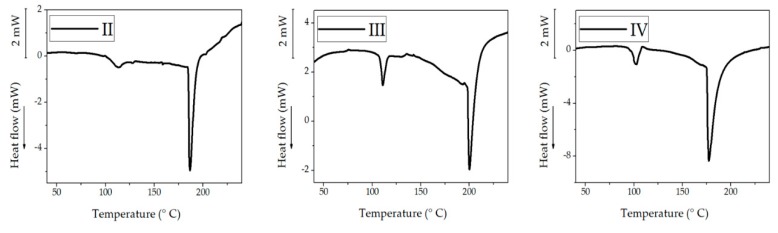
Thermograms for DM obtained from four different protocols.

The thermograms of the decellularized membranes of protocols II, III, and IV are shown in Figure 9, and they correspond to the first heating scan. It was not possible to obtain the thermogram from the DM of protocol I because of the sample decomposition. Different endothermic denaturation peaks may be seen viz.: II, 185 °C; III, 200 °C; and IV, 152 °C. Collagen materials exposed to high temperatures endure irreversible denaturation process [81,82,83,84,85,86]. Previous thermo-analytical studies of denaturation of collagen report that the denaturation temperature for bovine skin is 55 °C [87], bovine intramuscular connective tissue 90 °C [88], rat tail collagen 65 °C [89], bovine skin 50–55 °C [84], and type I collagen from bovine skin soluble in acid 117 °C [90]. As the water content is higher, collagen denaturation temperature gets higher, and this phenomenon may be observed in this study. It should be noted that collagen denatures; therefore, there was no second heating scan; furthermore the treated samples also showed another endothermic peak (105–115 °C) which is most likely related to gelatinous structures by the denaturation that were obtained at day 21 of culture for protocols II and III [84].

## 4. Conclusions

Decellularized membranes have attracted the attention of the scientific community since through tissue engineering it is possible to develop biological scaffolds that aim to deliver cells and proteins to damaged tissue, and at the same time, gradually degrade to make room for regenerated tissue. Within this frame, BAM were decellularized using four different protocols and the differences in terms of decellularization can be considered as negligible. All membranes obtained DNA concentrations <50 ng/mg, indicating that traces of the nucleic acid were present in the prepared material, although the obtained values are negligible which implies that DM do not have presence of native cells from the BAM. Nonetheless, protocol II proved to be the best method in terms of eliminating DNA content.

In the biological test, the obtained matrices from BAM were not cytotoxic for the cells (confirmed by MTT) and consisted of a source of collagen for recellularization. The mammalian skin cells adhered and conducted a remodeling effect on the BAM.

Each protocol may damage the ultrastructure of the tissue in different grade, mainly related to the chemical substances that were used. The extent of denaturation depends upon the interaction of the chemical substances with the molecules present in the tissue, and this analysis was supported by spectroscopic, thermal and topographical techniques.

Results showed that protocol IV (SDS 0.1%, NaOH 0.1 M, PAA + ascorbic acid 0.1, ethanol 70%, and PBS) could efficiently remove the cellular contents and preserve the native ECM architecture (confirmed for FTIR-ATR spectroscopy). Therefore, double layer bovine amniotic membranes (fetal and maternal) retained its biochemical properties after decellularization in comparison with the other membranes. Moreover, the mentioned double layer membrane exhibits a very low DNA concentration which is below to 50 ng/mg; for this reason, DM of protocol IV might be used as a possible biological substitute for skin.

The membranes of protocols I, II, and III, being single layer (stromal), had a greater surface area of exposure to the chemical agents used and, therefore, degraded further in terms of their composition. However, degradation was observed in culture, a semi-transparent gel was formed that may have potential biomedical applications, which may be part of later studies, underlining the potential applications of this matrices for tissue engineering.

The in vitro biocompatibility was demonstrated in this study, and it is indeed of pivotal importance, since this matrix may be considered as a potential source for the regeneration of epithelial tissue.

## Figures and Tables

**Table 1:** Decellularization protocols for the bovine amniotic membranes (BAM).

No.	Protocols
**I**	SDS 0.1% for 4 h NaOH 0.1 M for 1 h PAA + ascorbic acid 0.1 for 12 h Ethanol 70% for 1 h PBS for 2 h
**II**	SDS 0.1% for 4 h NaOH 0.1 M for 1 h PAA 0.15% + EtOH for 12 h NaOH 0.1 M for 1 h PAA for 1 h Ethanol 70% for 1 h PBS for 2 h
**III**	Tween 80 for 4 h NaOH 0.1 M for 1 h, PAA + ascorbic acid 0.1 for 12 h Ethanol 70% for 1 h PBS for 2 h
**IV**	Tween 80 for 4 h NaOH 0.1 M for 1 h PAA 0.15% + EtOH for 12 h NaOH 0.1 M for 1 h PAA for 1 h Ethanol 70% for 1 h PBS for 2 h
